# Location, Location, Location: an Antidote That Both Activates and Neutralizes a Toxin Used in Bacterial Warfare

**DOI:** 10.1128/jb.00161-23

**Published:** 2023-05-31

**Authors:** Shehryar Ahmad, John C. Whitney

**Affiliations:** a Temerty Faculty of Medicine, University of Toronto, Toronto, Ontario, Canada; b Department of Biochemistry and Biomedical Sciences, McMaster University, Hamilton, Ontario Canada; c Institute for Infectious Disease Research, McMaster University, Hamilton, Ontario, Canada; University of Chicago

**Keywords:** type VI secretion system, immunity proteins, Gram-negative bacteria, bacterial antagonism

## Abstract

S.J. Jensen, Z.C. Ruhe, A.F. Williams, D.Q. Nhan, et al. (J Bacteriol 205:e00113-23, 2023, https://doi.org/10.1128/jb.00113-23) demonstrate that a type VI secretion system (T6SS) immunity protein, Tli, functions to both neutralize and activate its cognate toxin, Tle, in Enterobacter cloacae. Their results reveal the surprising finding that Tli function differs, depending on its subcellular localization. Overall, this study enhances our understanding of T6SS immunity proteins, which are commonly viewed as monofunctional toxin-neutralizing antidotes.

## TEXT

The type VI secretion system (T6SS) is a protein nanomachine that mediates antagonism between contacting Gram-negative bacteria. To inhibit the growth of neighboring competitors, attacker cells “inject” a payload of toxic effector proteins into recipient cells in a T6SS-dependent manner. Effector proteins target conserved cellular structures and metabolites in the cytoplasm and periplasm, including cell membranes, the cell wall, nucleic acids, and nucleotides (1, 2). To prevent self-intoxication, T6SS effector genes are cotranscribed with genes encoding cognate immunity proteins, which typically function by directly occluding the toxin active site (3). During T6SS-mediated export, effectors dissociate from immunity proteins in the cytoplasm and are delivered into recipient cells as active toxins (4). Consequently, immunity genes for effectors that act in the cytoplasm are essential for the viability of toxin-producing cells (5, 6). In contrast, early work in the T6SS field demonstrated that immunity genes associated with periplasm-targeting effectors could be mutationally inactivated without impacting the fitness of effector-producing cells if these cells are grown under conditions that are not conducive to T6SS-dependent intercellular intoxication (e.g., growth in liquid media) (7–9). Furthermore, the observation that immunity mutants can succumb to intercellular intoxication demonstrates that effector activity and delivery via the T6SS are unimpaired. Based on these findings, the consensus has remained that immunity proteins exclusively function as toxin-neutralizing antidotes that confer protection against effectors.

**FIG 1:**
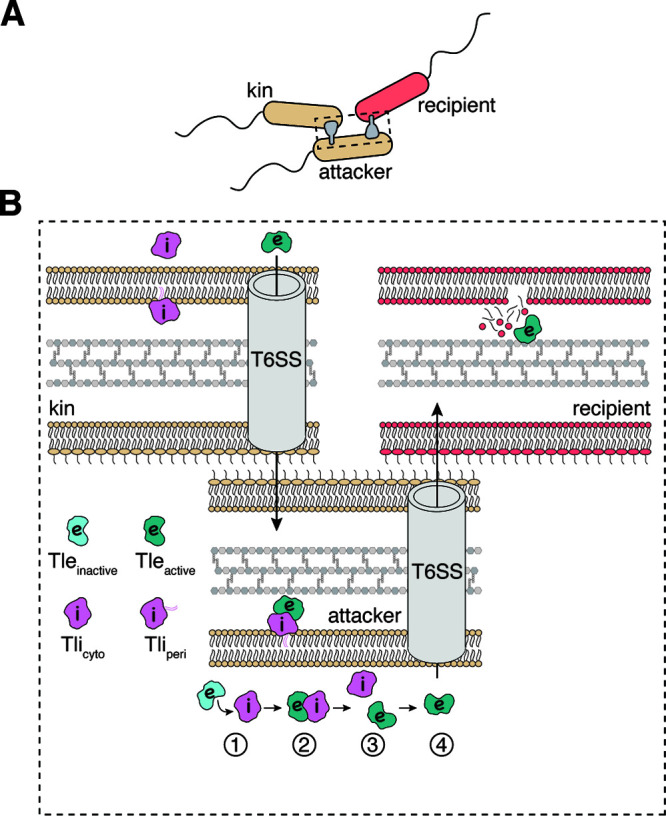
The function of Tli depends on its subcellular localization. (A) In coculture competition, an attacker cell (beige) uses its type VI secretion system (T6SS; shown in gray) to “inject” effectors into a recipient cell to inhibit the growth of a competitor (red). In some instances, the attacker cells are attacked by kin cells (labeled “kin”; beige), which is a neutral interaction due to the presence of immunity proteins and therefore does not result in growth inhibition. (B) Tli exists either as a lipoprotein in the periplasm (labeled Tli_peri_; pink) or as a soluble protein in the cytoplasm (labeled Tli_cyto_; pink) due to alternative start sites downstream of the lipoprotein signal sequence present within the Tli primary sequence. Four steps take place within the attacker cell before Tle is exported: (i) inactive Tle (light blue) binds Tli_cyto_ in the cytoplasm; (ii) the interaction of Tle with Tli_cyto_ results in the activation of the effector via an unknown mechanism (depicted as a changing color to dark blue) (iii) the activated Tle and Tli_cyto_ dissociate from one another as the effector is recruited to the T6SS apparatus; and (iv) the active Tle is exported into a recipient cell and inhibits growth through its phospholipase activity. In addition to the events depicted in the attacker cell, other neighboring kin cells may inject the attacker with activated Tle, which does not cause growth inhibition, as periplasmic Tli_peri_ immunity inhibits its toxic phospholipase activity.

In this issue of the J Bacteriol, Jensen et al. (10) report findings that extend our current understanding of T6SS immunity protein function through their analysis of the Tle-Tli effector-immunity pair from Enterobacter cloacae. Using a series of mutagenesis experiments and coculture competition assays, the authors reveal that the Tli immunity protein exhibits dual functionality, depending on its subcellular localization. In the periplasm, Tli confers protection against incoming Tle toxin from neighboring cells, whereas in the cytoplasm, Tli is paradoxically required to activate Tle prior to its T6SS-dependent export (Fig. 1).

Interest in characterizing Tle and Tli came from the results of a genetic screen that Jensen et al. (10) designed to study another interbacterial antagonism pathway called contact-dependent inhibition (CDI) (11, 12). The CDI pathway of E. cloacae is inactive when bacteria are grown under standard culturing conditions. Therefore, the authors were interested in identifying transcriptional regulators that may be repressing this pathway. A strain of E. cloacae expressing a glucuronidase-encoding reporter gene fused to the *cdi* promoter was subjected to transposon mutagenesis screening to identify potential regulators. To their surprise, the isolated mutants had no effect on the expression levels of the CDI pathway but instead had increased permeability to the glucuronidase substrate X-gluc. The majority of the transposon insertion sites mapped to the *tli* gene, which encodes the ankyrin repeat-containing Tli immunity protein that has previously been shown to neutralize the periplasm-targeting phospholipase T6SS effector Tle (13). These initial findings led the authors to speculate that the observed hyperpermeability phenotype is due to an unopposed Tle phospholipase activity causing the perturbation of the cell membranes. Indeed, when a plasmid expressing wild-type *tli* was introduced into the mutant strains, they no longer displayed a permeability defect. The authors further demonstrated that hyperpermeability is T6SS-dependent and therefore occurs due to the delivery of Tle from neighboring cells.

Tli possesses an N-terminal lipoprotein signal sequence that directs its localization to the periplasm so that it can protect against incoming Tle injections by neighboring cells, which is a common property of immunity proteins for periplasm-targeting effectors (7–9). The majority of the transposon insertions that were identified in *tli* by Jensen et al. (10) were within this lipoprotein signal sequence region, and the authors showed that, in contrast to the wild-type protein, these transposon-disrupted variants of Tli are not exported across the inner membrane to the periplasm and thus cannot confer protection against periplasm-delivered Tle. In addition to the insertion mutants that were present within the N-terminal lipoprotein region of Tli, Jensen et al. (10) notably observed that mutants with insertion sites downstream of the lipoprotein region exhibited more modest permeability to X-gluc. This is particularly striking, as one would expect insertions in the *tli* gene that disrupt the mature form of the protein to be equally detrimental, if not more detrimental, to immunity function. The authors extended these findings to show that a Δ*tli* mutant also does not exhibit hyperpermeability, despite it expressing Tle to wild-type levels. Furthermore, E. cloacae strains containing Tli insertion mutations within the lipoprotein signal sequence exhibit a competitive advantage in coculture competition assays against Tle-sensitive recipients, whereas mutants with insertion sites downstream of this region do not. This suggests that cytoplasmic Tli is functional and necessary for Tle-mediated growth inhibition.

These findings led the authors to suspect that Tli may be required for the recruitment of Tle to the T6SS apparatus and/or may be required to activate Tle prior to export. Whereas the former was not addressed directly, Jensen et al. (10) fused the lipase domain of Tle to the secreted T6SS spike protein, VgrG2, to ensure that the lipase would be delivered into the target cells. Notably, coculture competition assays with chimeric VgrG2-lipase showed that this fusion is only toxic if coexpressed with Tli. This finding is quite interesting, as it demonstrates that even when effector export is ensured, the immunity-effector interaction is still required to facilitate the growth inhibition of target cells.

Our current understanding of the role of T6SS immunity proteins in effector stability and export stems from an analysis by Li et al. on the Tse2-Tsi2 effector-immunity pair from Pseudomonas aeruginosa (4, 14). These authors showed that Tsi2 stabilizes Tse2 in the cytoplasm but that this stabilizing effect is not required for effector export through the T6SS apparatus. Tse2 is a cytoplasm-targeting effector. Therefore, the analyses conducted by Li et al. necessitated the use of a nontoxic Tse2 variant to generate an immunity-deficient strain. Consequently, it was not possible to assess the effect of immunity interaction on effector activation. In the current study by Jensen et al., the authors demonstrate that the wild-type Tle effector is stable in the absence of its cognate immunity but cannot inhibit target cell growth, regardless of whether export is ensured. To our knowledge, this is the first study to show that the genetic perturbation of an immunity protein impacts the toxic activity of its cognate effector.

The discovery made by Jensen et al. (10) provides an important starting point for the further examination of the additional functions of T6SS immunity proteins, beyond toxin neutralization, and raises several interesting questions. First, how does the T6SS apparatus itself impact the Tle-Tli interaction? Tle presumably dissociates from cytosolic Tli after its activation and prior to its export, but the mechanism for this remains unknown. Future experiments involving the heterologous expression of periplasm-directed Tle in the presence or absence of Tli may help ascertain whether the T6SS apparatus itself is necessary for Tle activation. Second, how is Tle activation maintained during a T6SS injection event after Tli dissociation occurs? If interaction with cytoplasmic Tli induces a conformational change in Tle that makes it enzymatically active, it is unclear how this conformation would be maintained during the Tle transit of the T6SS apparatus. Finally, what is the molecular basis for Tle activation by Tli? The authors’ findings suggest that there may exist Tli mutants that prevent the activation of the toxin. An in-depth analysis of the specific contacts between Tle and Tli, coupled with approaches such as the random mutagenesis of Tli, may uncover important residues that are necessary for Tle activation. Previous studies suggest that the immunity-dependent activation of effectors is likely not generalizable, as the immunity mutants of several phospholipase and amidase effectors that have been previously characterized by Russell et al. deliver active effectors between adjacent cells via the T6SS (7, 8). Thus, it will be interesting to see how widespread the immunity-dependent activation of effectors is among the many T6SS effectors that have been identified to date.

## References

[1] Russell AB, Peterson SB, Mougous JD. 2014. Type VI secretion system effectors: poisons with a purpose. Nat Rev Microbiol 12:137–148. doi:10.1038/nrmicro3185. 24384601 PMC4256078

[2] Klein TA, Ahmad S, Whitney JC. 2020. Contact-dependent interbacterial antagonism mediated by protein secretion machines. Trends Microbiol 28:387–400. doi:10.1016/j.tim.2020.01.003. 32298616

[3] Durand E, Cambillau C, Cascales E, Journet L. 2014. VgrG, Tae, Tle, and beyond: the versatile arsenal of Type VI secretion effectors. Trends Microbiol 22:498–507. doi:10.1016/j.tim.2014.06.004. 25042941

[4] Li M, Le Trong I, Carl MA, Larson ET, Chou S, De Leon JA, Dove SL, Stenkamp RE, Mougous JD. 2012. Structural basis for type VI secretion effector recognition by a cognate immunity protein. PLoS Pathog 8:e1002613. doi:10.1371/journal.ppat.1002613. 22511866 PMC3325213

[5] Whitney JC, Quentin D, Sawai S, LeRoux M, Harding BN, Ledvina HE, Tran BQ, Robinson H, Goo YA, Goodlett DR, Raunser S, Mougous JD. 2015. An interbacterial NAD(P)(+) glycohydrolase toxin requires elongation factor Tu for delivery to target cells. Cell 163:607–619. doi:10.1016/j.cell.2015.09.027. 26456113 PMC4624332

[6] Ahmad S, Wang B, Walker MD, Tran HR, Stogios PJ, Savchenko A, Grant RA, McArthur AG, Laub MT, Whitney JC. 2019. An interbacterial toxin inhibits target cell growth by synthesizing (p)ppApp. Nature 575:674–678. doi:10.1038/s41586-019-1735-9. 31695193 PMC6883173

[7] Russell AB, Hood RD, Bui NK, LeRoux M, Vollmer W, Mougous JD. 2011. Type VI secretion delivers bacteriolytic effectors to target cells. Nature 475:343–347. doi:10.1038/nature10244. 21776080 PMC3146020

[8] Russell AB, LeRoux M, Hathazi K, Agnello DM, Ishikawa T, Wiggins PA, Wai SN, Mougous JD. 2013. Diverse type VI secretion phospholipases are functionally plastic antibacterial effectors. Nature 496:508–512. doi:10.1038/nature12074. 23552891 PMC3652678

[9] Wood TE, Howard SA, Forster A, Nolan LM, Manoli E, Bullen NP, Yau HCL, Hachani A, Hayward RD, Whitney JC, Vollmer W, Freemont PS, Filloux A. 2019. The Pseudomonas aeruginosa T6SS delivers a periplasmic toxin that disrupts bacterial cell morphology. Cell Rep 29:187–201. doi:10.1016/j.celrep.2019.08.094. 31577948 PMC6899460

[10] Jensen SJ, Ruhe ZC, Williams AF, Nhan DQ, Garza-Sánchez F, Low DA, Hayes CS. 2023. Paradoxical activation of a type VI secretion system phospholipase effector by its cognate immunity protein. J Bacteriol 205:e00113-23. doi:10.1128/jb.00113-23. PMC10294671 37212679

[11] Hayes CS, Aoki SK, Low DA. 2010. Bacterial contact-dependent delivery systems. Annu Rev Genet 44:71–90. doi:10.1146/annurev.genet.42.110807.091449. 21047256

[12] Aoki SK, Pamma R, Hernday AD, Bickham JE, Braaten BA, Low DA. 2005. Contact-dependent inhibition of growth in Escherichia coli. Science 309:1245–1248. doi:10.1126/science.1115109. 16109881

[13] Donato SL, Beck CM, Garza-Sanchez F, Jensen SJ, Ruhe ZC, Cunningham DA, Singleton I, Low DA, Hayes CS. 2020. The beta-encapsulation cage of rearrangement hotspot (Rhs) effectors is required for type VI secretion. Proc Natl Acad Sci USA 117:33540–33548. doi:10.1073/pnas.1919350117. 33323487 PMC7777165

[14] Robb CS, Robb M, Nano FE, Boraston AB. 2016. The structure of the toxin and type six secretion system substrate Tse2 in complex with its immunity protein. Structure 24:277–284. doi:10.1016/j.str.2015.11.012. 26749446

